# Application and comparison of ARIMA, LSTM, and ARIMA-LSTM models for predicting foodborne diseases in Liaoning Province

**DOI:** 10.3389/fdata.2025.1666962

**Published:** 2025-11-12

**Authors:** Xiaoxiao Du, Haomiao Yu, Hao Zhang, Xiangyun Liu, Xinling Yu, Tao Xie, Wenli Diao

**Affiliations:** Liaoning Provincial Center for Disease Control and Prevent, Shenyang, Liaoning, China

**Keywords:** foodborne, ARIMA model, LSTM model, ARIMA-LSTM model, predicting

## Abstract

**Objective:**

To compare the application of the ARIMA model, the Long Short-Term Memory (LSTM) model and the ARIMA-LSTM model in forecasting foodborne disease incidence.

**Methods:**

Monthly case data of foodborne diseases in Liaoning Province from January 2015 to December 2023 were used to construct ARIMA, LSTM, and ARIMA-LSTM models. These three models were then applied to forecast the monthly incidence of foodborne diseases in 2024, and their predictions were compared with those of a baseline model. Model performance was evaluated by comparing the predicted and observed values using root mean square error (RMSE), mean absolute error (MAE), and mean absolute percentage error (MAPE), allowing identification of the optimal model. The best-performing model was subsequently employed to predict the monthly incidence for 2025.

**Results:**

The ARIMA-LSTM model was identified as the optimal model. Specifically, the ARIMA (2,0,0) (0,1,1)1_2_ model produced RMSE = 300.03, MAE = 187.11, and MAPE = 16.38%, while the LSTM model yielded RMSE = 408.71, MAE = 226.03, and MAPE = 17.21%. In contrast, the ARIMA-LSTM model achieved RMSE = 0.44, MAE = 0.44, and MAPE = 0.08%, representing a dramatic improvement over the baseline model (RMSE = 204.17, MAE = 146.75, MAPE = 15.62%), with reductions of 99.5%, 99.7%, and 99.4% in RMSE, MAE, and MAPE, respectively. Based on the ARIMA–LSTM model, the predicted monthly cases of foodborne diseases for 2025 are: 214.62 (Jan), 260.84 (Feb), 462.92 (Mar), 590.92 (Apr), 800.88 (May), 965.11 (Jun), 2410.36 (Jul), 2651.36 (Aug), 1711.15 (Sep), 941.22 (Oct), 628.21 (Nov), and 465.05 (Dec).

**Conclusion:**

The ARIMA-LSTM model is considered the optimal model for predicting foodborne disease incidence in Liaoning Province in 2025.

## 1 Introduction

Foodborne diseases represent one of the most critical global public health challenges, significantly impacting human health and quality of life (Ntshoe et al., 2021). According to World Health Organization (WHO) data, millions of people worldwide fall ill or die annually due to foodborne illnesses. In 2015, the WHO released its first global estimates of foodborne disease burden, revealing that nearly 1 in 10 people globally suffer from illnesses caused by contaminated food each year, resulting in 420,000 deaths and the loss of 33 million healthy life-years (DALYs) (Kirk et al., 2015).

Foodborne diseases exhibit notable seasonal patterns (Yu et al., 2024). Therefore, developing predictive models based on historical incidence data can provide valuable support for the prevention and control of foodborne diseases. The ARIMA model is a commonly used method in infectious disease forecasting and is particularly well-suited for seasonal data. However, foodborne disease incidence is also influenced by factors such as geography, climate, and socioeconomic conditions, leading to nonlinear trends (Siguo et al., 2022; Pijnacker et al., 2024). The LSTM model, with its capacity for nonlinear fitting and its ability to capture temporal patterns in data, may improve forecasting accuracy (Gao et al., 2025). The ARIMA model performs well in capturing linear trends and seasonality (Balawi and Tenekeci, 2024); however, it assumes that the data-generating process is primarily linear, making it difficult to model complex nonlinear dynamics (Sattarzadeh et al., 2025). In recent years, Long Short-Term Memory (LSTM) networks have demonstrated strong capabilities in time series forecasting, particularly in handling long-term dependencies and nonlinear features (Mahmoudi, 2025). Nevertheless, using LSTM alone also has limitations, such as requiring a relatively large amount of training data and being less efficient in modeling linear components (Lim and Zohren, 2021). To overcome the shortcomings of individual models, researchers have proposed the ARIMA–LSTM hybrid model (Ray et al., 2023).

In this study, we constructed ARIMA, LSTM, and ARIMA-LSTM hybrid models using foodborne disease data from 2015 to 2023. These three models were employed to predict the monthly number of foodborne disease cases in 2024, and the predictions were compared with the actual reported cases as well as with a baseline model to identify the most accurate model. Finally, the optimal model was applied to forecast the number of cases in 2025, providing a scientific basis for the development of prevention and control strategies for foodborne diseases in Liaoning Province.

## 2 Materials and methods

### 2.1 Data sources

Monthly case counts of foodborne diseases from January 2015 to December 2024 were extracted from the Liaoning Provincial Foodborne Disease Risk Surveillance System. The dataset from January 2015 to December 2023 served as the training set, while the data from 2024 were used as the validation set for model evaluation.

### 2.2 Research methods

#### 2.2.1 ARIMA model

**Figure 1:**
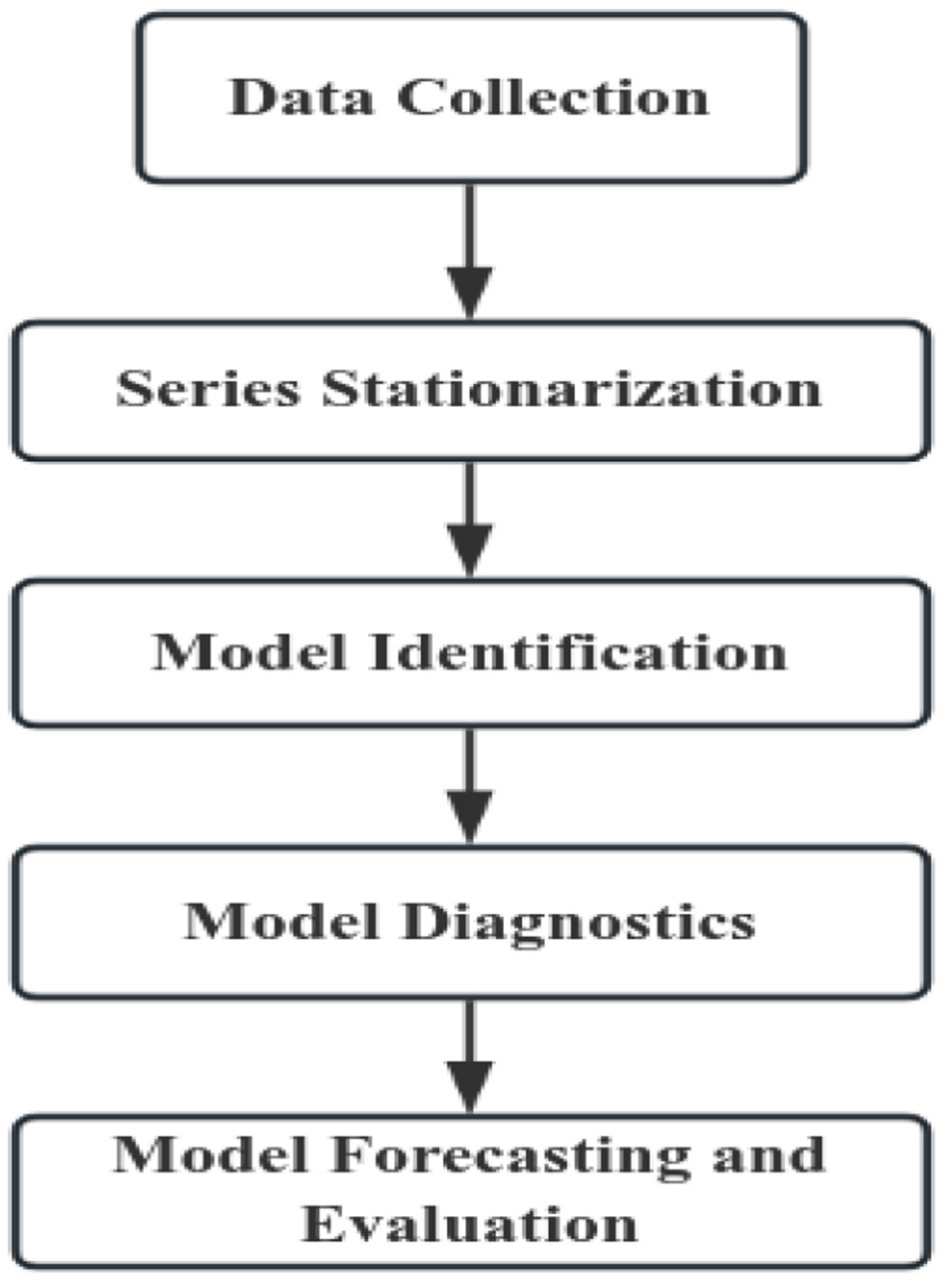
Flowchart of ARIMA model construction.

The construction process of the ARIMA model is shown in Figure 1.

The autoregressive integrated moving average (ARIMA) model is a linear, univariate time series model that combines three components: autoregression (AR), differencing (I), and moving average (MA) (Wagner and Cleland, 2023). Its general form can be expressed as:


$$\begin{array}{l} ARIMA\left(p,d,q\right):\;\varphi \left(B\right){\left(1-B\right)}^{d}yt=\theta \left(B\right)\varepsilon t \end{array}$$

Where *yt* denotes the observed value at time *t, B* is the backward shift operator *(Byt* = *y*_*t*−1_*)*, and *d* represents the order of differencing required to achieve stationarity. The autoregressive component is defined as φ*(B)*=*1–*φ_1_*B–*φ_2_*...B*^2^*-...–*φ_*p*_*B*^*p*^, while the moving average component is defined as θ*(B)*=*1*+θ_1_*B*+θ_2_*B*^2^+*..*.+θ_*q*_*B*^*q*^. The error term ε*t* represents white noise.

In practice, the differencing operator *(1–B)*^*d*^ is applied to remove non-stationarity in the series. The AR part captures the dependence of the current observation on its past values, whereas the MA part accounts for the dependency on past forecast errors. By integrating these three components, ARIMA provides a flexible yet interpretable framework for modeling and forecasting time series data.

Considering the seasonal characteristics of foodborne diseases, the model structure was specified as ARIMA (p,d,q) (P, D, Q)s, where:

- p and q represent the orders of non-seasonal autoregression and moving average, respectively;
- d denotes the degree of non-seasonal differencing;
- P and Q indicate the seasonal autoregressive and moving average orders;
- D stands for seasonal differencing;
- s corresponds to the seasonal period length.

##### 2.2.1.1 Series stationarization

Prior to modeling, the time series must be tested for stationarity using the ADF test. For non-stationary series, appropriate transformations (e.g., Box-Cox transformations, seasonal differencing) are applied until stationarity is confirmed via repeated testing.

##### 2.2.1.2 Model identification

The autocorrelation function (ACF) and partial autocorrelation function (PACF) plots are examined to determine the preliminary model structure. Based on the ACF and PACF patterns, initial parameters are identified (typically with orders not exceeding 2) (Peng, 2014). The Akaike Information Criterion (AIC) and Bayesian Information Criterion (BIC) are then computed, and the model with the lowest AIC and BIC values is selected as the optimal model.

##### 2.2.1.3 Model diagnostics

*Residual diagnostics* were conducted using the Ljung-Box test for white noise. The non-significant test result (*P* > 0.05) confirms the residuals are uncorrelated, validating the model's suitability for predictions.

##### 2.2.1.4 Model forecasting

The final selected optimal model was employed for prediction. The goodness-of-fit between the actual and predicted values across the entire series was evaluated using the RMSE, MAE, MAPE.

#### 2.2.2 LSTM model

The Long Short-Term Memory (LSTM) model is a specialized type of recurrent neural network (RNN) that incorporates memory cells along with three gating mechanisms—namely, the input gate, forget gate, and output gate (Aldrich et al., 2022). These gates regulate the flow of information and enable the model to selectively retain or discard information, thereby effectively addressing the issues of vanishing and exploding gradients commonly encountered in modeling long time series with traditional RNNs (Malashin et al., 2024).

**Figure 2:**
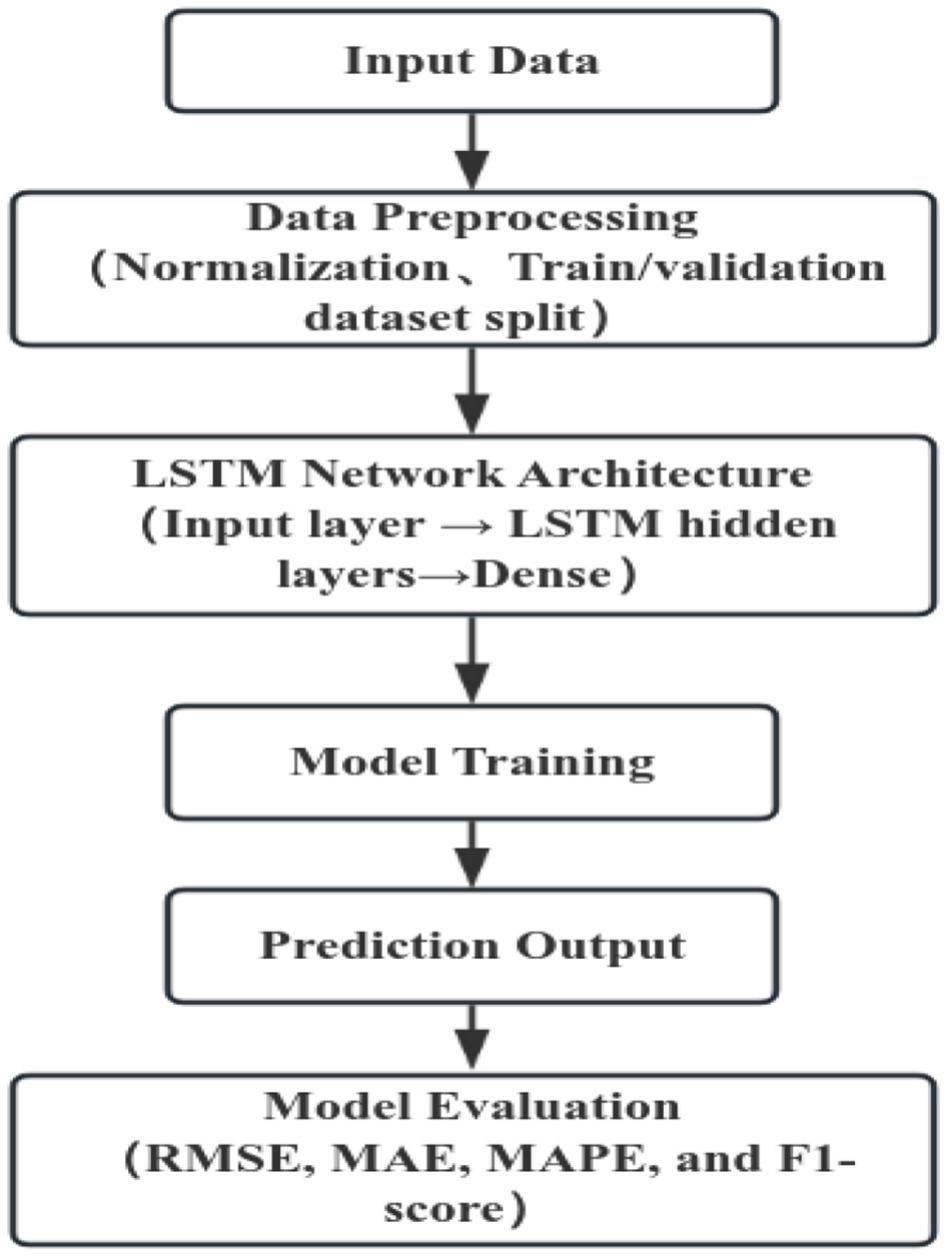
Flowchart of LSTM model development.

Prior to model construction, all time series data were normalized to the range [0, 1] using the Min–Max scaling method to ensure numerical stability during training. Input sequences were generated with a fixed time window, where the values from the preceding N time steps were used to predict the next observation. The dataset was divided into a training set and a validation set to assess model performance (Figure 2).

The long short-term memory (LSTM) network was implemented with the following architecture and configurations:

Hidden units: determining the model's capacity to capture temporal dependencies.

Number of LSTM layers: to balance model complexity and computational efficiency.

Dropout rate: a dropout rate of 0.2 was applied to mitigate overfitting.

Activation functions: tanh and sigmoid, as defined by the standard LSTM architecture.

Optimizer: Adam with a learning rate of 0.001.

Loss function: mean squared error (MSE), suitable for continuous-valued predictions.

Batch size: defined the number of samples.

Epochs: with an early stopping criterion to prevent overfitting, and early stopping strategy was employed based on the validation loss with a patience of 10 epochs.

#### 2.2.3 ARIMA-LSTM model

**Figure 3:**
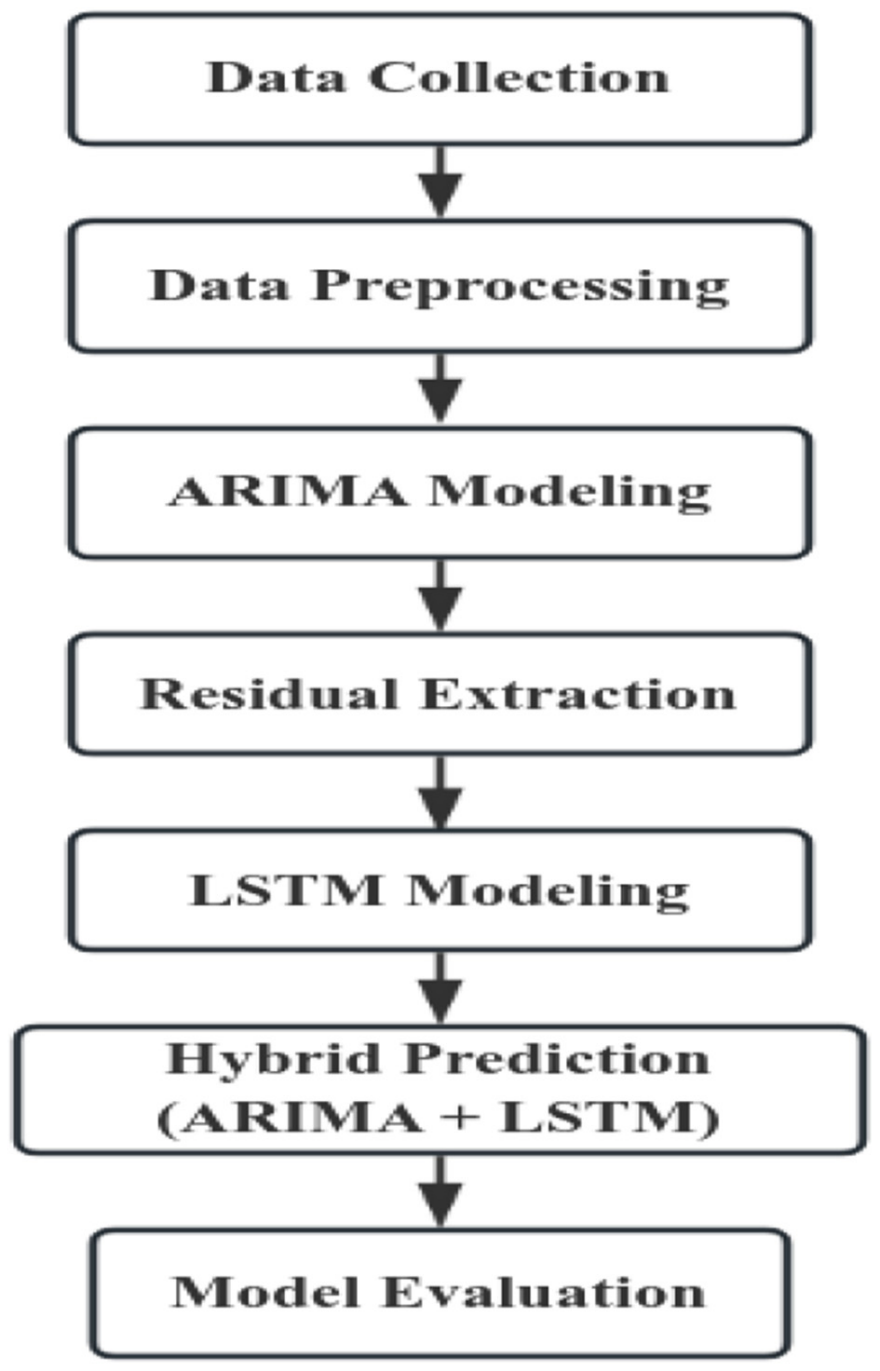
Flowchart of ARIMA–LSTM model construction.

The following is a simplified flowchart for constructing the ARIMA–LSTM model (Figure 3).

First, the collected data were organized and preprocessed. An ARIMA model was then constructed using the processed data to generate forecasts, which were compared with the observed values to obtain the residuals. The residuals were subsequently modeled with an LSTM network to capture their nonlinear characteristics, producing forecasts of the residual component. Finally, the predictions from the ARIMA model and the LSTM residual forecasts were combined to obtain the predictions of the hybrid model.

#### 2.2.4 Baseline model

Since foodborne diseases exhibit seasonality, the Seasonal Naïve method can be used as a baseline model. This approach is a simple yet commonly applied time series forecasting method for data with seasonal patterns, where the forecast value is set equal to the observation from the same point in the previous season.

ŷ_*t*_ = *y*_*t*−*s*_

Here, *s* is the length of the seasonal period, ŷ_*t*_ is the forecasted value, *y*_*t*−*s*_ and is the observed value from the same month in the previous year or the same quarter in the previous season.

#### 2.2.5 Model evaluation metrics

In this study, model performance in terms of fitting and forecasting was assessed using RMSE, MAE, MAPE value of less than 10% indicates excellent predictive performance, values between 10% and 20% indicate good performance, and values below 40% are considered acceptable.

#### 2.2.6 Statistical methods

Data were organized using Excel 2019. The ARIMA model was constructed using R 4.4.2 software, while the LSTM model and the ARIMA-LSTM model were developed using Python 3.13. A significance level of α = 0.05 was adopted, and *P*-values less than 0.05 were considered statistically significant.

## 3 Results

### 3.1 ARIMA model construction

#### 3.1.1 Time series stationarity

**Figure 4:**
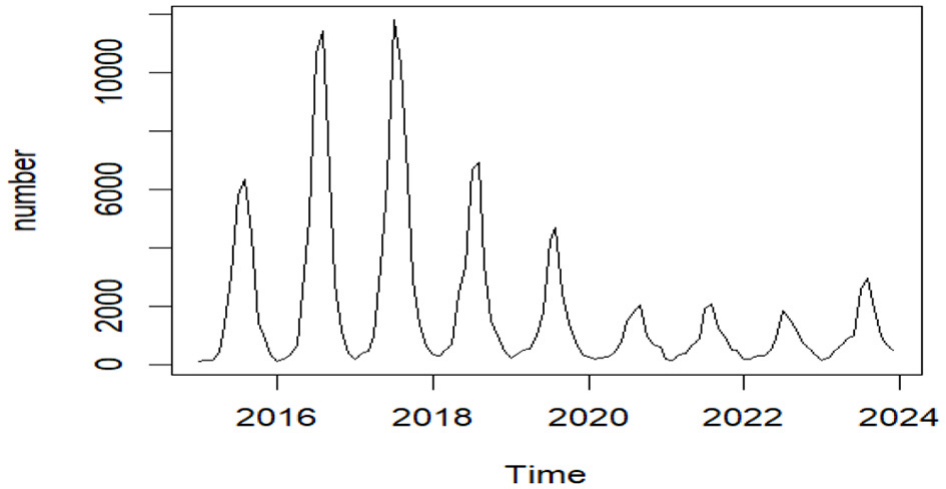
The epidemiological trend of reported foodborne disease cases in Liaoning Province, 2015–2023.

The stationarity of the raw time series data (Figure 4) was examined through the Augmented Dickey-Fuller test (ADF). A statistically significant *p*-value (*P* = 0.01 < 0.05) suggested the rejection of the null hypothesis, demonstrating that the data followed a stationary time series pattern; therefore, *d* = 0.

#### 3.1.2 Model identification and order determination

**Figure 5:**
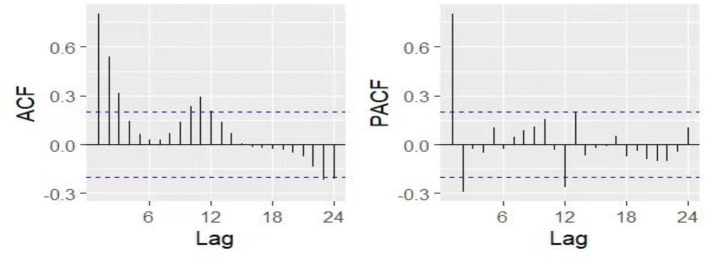
The ACF and PACF plots after first-order seasonal differencing. ACF, autocorrelation function; PACF, partial autocorrelation function.

Seasonality was removed by applying seasonal differencing identified through *nsdiffs()* function, resulting in D = 1. Following the principle of model parsimony and practical experience, the orders of p, q, P, and Q were generally limited to no more than 2. Examination of the ACF and PACF plots (Figure 5), together with parameter optimization using the *auto.arima* function from the R *forecast* package and consideration of AIC/BIC minimization criteria, led to the selection of ARIMA (2,0,0) (0,1,1)1_2_ as the optimal model (AICc = 1,548.26).

#### 3.1.3 Model diagnostics

**Figure 6:**
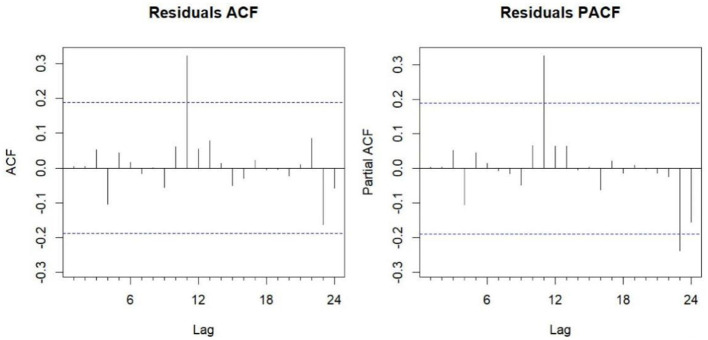
ACF and PACF plots of the residual series from the fitted ARIMA (2,0,0) (0,1,1)_12_ model. ACF, autocorrelation function; PACF, partial autocorrelation function.

The Box-Ljung test for white noise produced a *p*-value of 0.9696 (*P* > 0.05), confirming that the model residuals exhibit white noise behavior, suggesting all extractable information from the time series has been captured. The autocorrelation (ACF) and partial autocorrelation (PACF) plots of the residual series (Figure 6) show that nearly all residuals fall within the 95% confidence intervals, demonstrating the model's adequacy for forecasting.

#### 3.1.4 Model fitting

**Figure 7:**
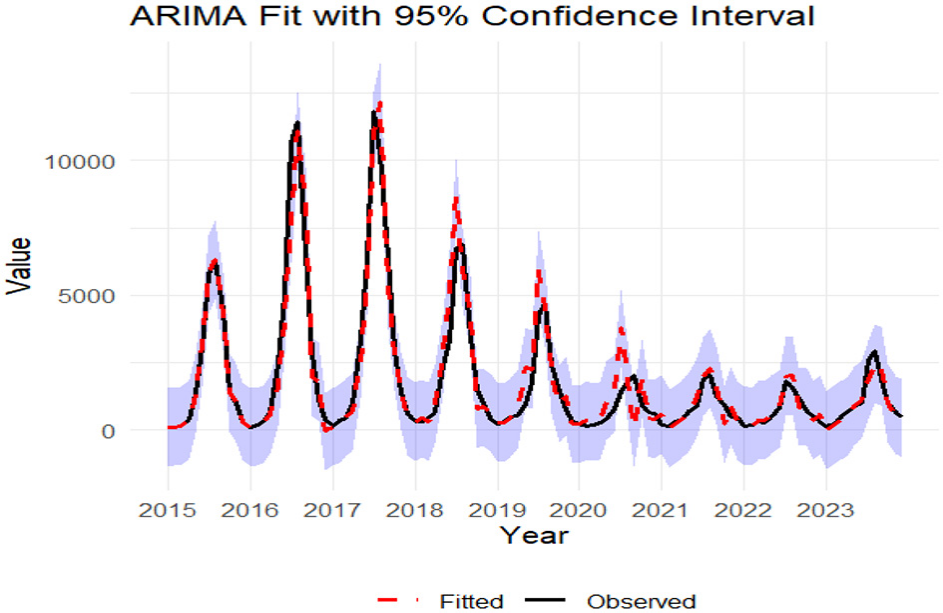
The epidemiological trends of observed vs. fitted data for foodborne diseases (2015-2023). The shaded zone represents the 95% confidence interval of the fitted values.

The monthly incidence of foodborne diseases from 2015 to 2023 was fitted using a seasonal ARIMA (2,0,0) (0,1,1)1_2_ model. The fitted values exhibited strong agreement with the observed data trends (Figure 7). The model was then applied to forecast case numbers for January to December 2024 (Table 1).

**Table 1:** Comparison between predicted and observed monthly incidence of foodborne diseases in 2024 (case).

**Time**	**Predicted values**	**Actual values**	**95% LCL**	**95% UCL**
Jan	231.81	277	−1,217.18	1,680.80
Feb	274.95	182	−1,901.10	2,451.00
Mar	470.81	421	−2,061.32	3,002.94
Apr	594.76	593	−2,080.54	3,270.06
May	802.44	845	−1,919.01	3,523.89
Jun	965.57	1,281	−1,767.05	3,698.19
Jul	2,410.42	2,848	−323.88	5,144.72
Aug	2,651.36	3,484	−83.00	5,385.73
Sept	1,711.24	1,739	−1,023.16	4,445.64
Oct	841.42	1,108	−1,793.07	3,675.92
Nov	628.51	683	−2,106.06	3,363.08
Dec	465.42	544	−2,269.19	3,200.02

LCL, Lower confidence limit; UCL, Upper confidence limit.

### 3.2 LSTM model development

**Figure 8:**
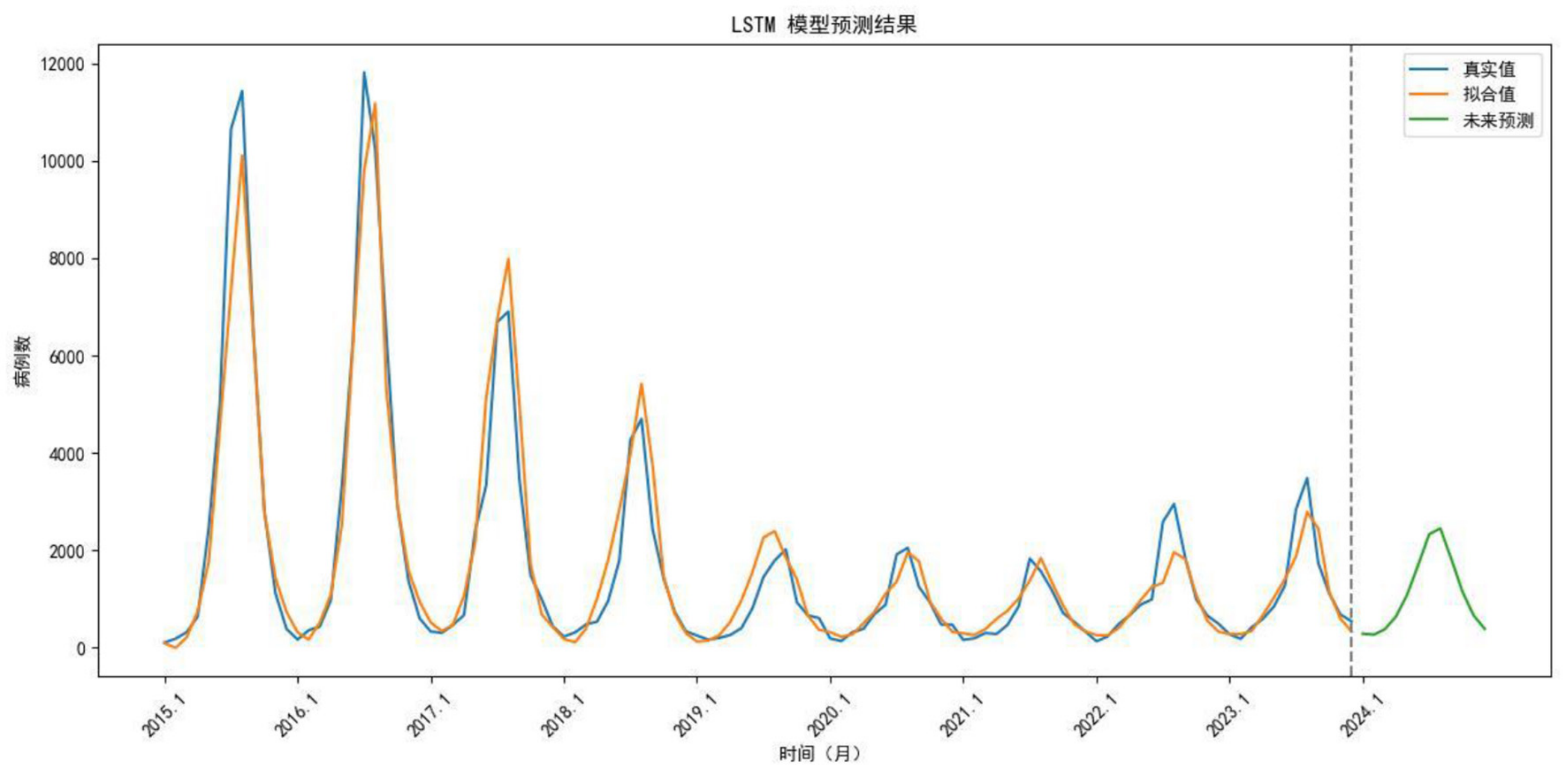
Comparison of trends between fitted and actual values from 2015 to 2023, as well as the forecast for 2024. Blue line, True value; Orange line, Fitted value; Green line, Predicted value.

An LSTM model was constructed using data from 2015 to 2023 as the training set and data from 2024 as the validation set. The data were normalized to a range between 0 and 1 prior to model construction. Given the seasonal characteristics of the dataset, a time step of 12 was selected as optimal. For a comprehensive consideration of avoiding overfitting, maintaining computational efficiency, and ensuring interpretability, the LSTM model consists of two layers: an LSTM layer and a Dense layer, and each with 50 hidden units. The Adam optimizer was employed, with mean squared error (MSE) used as the loss function. The Adam optimizer was selected due to its adaptive learning rate mechanism, fast convergence, and robustness, which make it a widely adopted and effective choice for time series prediction tasks. The batch size was set to 12. The model was trained for 200 epochs. After training, both the fitted and predicted values were denormalized (Figure 8). Based on this model, monthly case numbers for 2024 were forecasted and compared with the observed values (Table 2).

**Table 2:** Comparison between predicted and actual values for 2024 (case).

**Time**	**Predicted values**	**Actual values**
Jan	300.35	227
Feb	258.13	182
Mar	323.40	421
Apr	520.96	593
May	896.40	845
Jun	1,470.41	1,281
Jul	2,129.49	2,848
Aug	2,302.45	3,484
Sept	1,765.14	1,739
Oct	1,168.87	1,108
Nov	718.35	683
Dec	445.62	544

### 3.3 ARIMA-LSTM model construction

**Figure 9:**
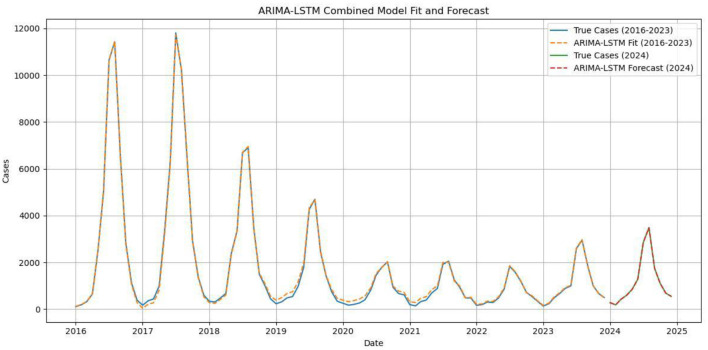
Comparison of the ARIMA–LSTM model fitted values with the actual values for 2016–2023, and comparison of the predicted and actual values for 2024.

Since the ARIMA forecasts had already been obtained, the corresponding residuals were calculated and used to build an LSTM model. Residuals from 2015 to 2023 were used for training, and those from 2024 for validation. To reduce overfitting while maintaining efficiency, the model included one LSTM layer and one Dense layer, with 64 hidden units. A time step of 12 was selected to account for the seasonal pattern in the residuals. The tanh activation function, default in LSTM and suitable for nonlinear time series, was applied, while the Adam optimizer was chosen for its adaptive and efficient learning. Given the relatively small dataset (120 observations), a batch size of 8 and 100 epochs were used. The residual predictions were then combined with the ARIMA forecasts to generate the final results, which showed close agreement with the observed values (Figure 9). The specific forecasted values for 2024 are shown in Table 3.

**Table 3:** Comparison between the ARIMA–LSTM model forecasts and the actual values for 2024 (case).

**Time**	**Predicted values**	**Actual values**
Jan	276.56	277
Feb	181.56	182
Mar	420.56	421
Apr	592.56	593
May	844.56	845
Jun	1,280.55	1,281
Jul	2,847.56	2,848
Aug	3,834.56	3,484
Sept	1,738.56	1,739
Oct	1,107.56	1,108
Nov	682.56	683
Dec	543.56	544

### 3.4 Baseline model development

Because foodborne diseases exhibit seasonal periodicity, the parameter was set to *s* = 12, and the baseline model forecasts (ŷ_*t*_)for 2024 are equal to the actual values of 2023.

### 3.5 Comparison of forecasting performance between the four models

**Figure 10:**
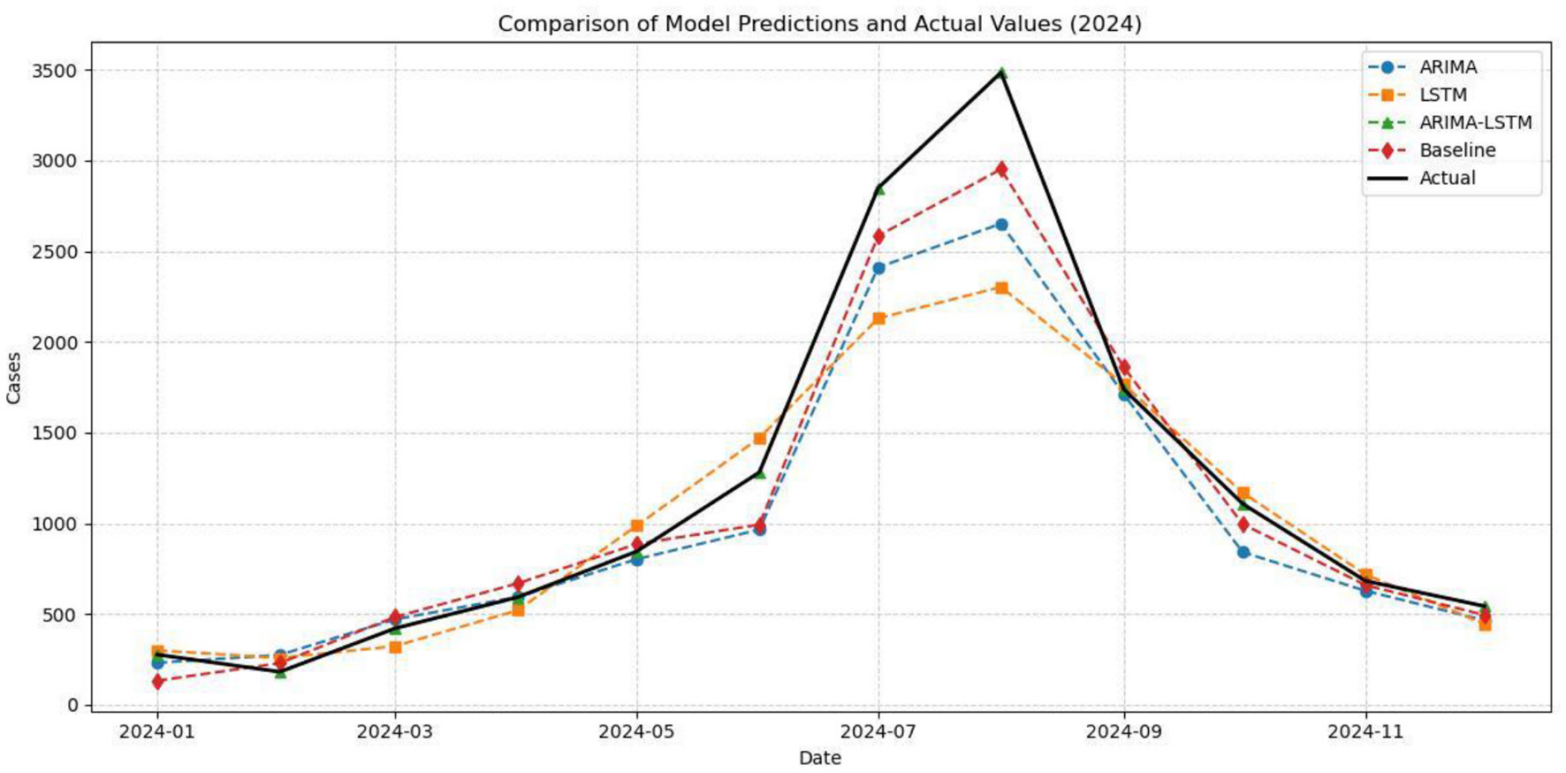
Comparison of the 2024 forecasts from the four models with the actual values in 2024.

The predicted values of the four models for 2024 were compared with the actual observations, and model performance was evaluated using RMSE, MAE, and MAPE (Figure 10). The results showed that the ARIMA-LSTM hybrid model achieved the best agreement with the observed data and had the lowest error metrics (Table 4). Compared with the baseline model, RMSE, MAE, and MAPE were reduced by 99.5%, 99.7%, and 99.4%, respectively. Therefore, the ARIMA-LSTM model was identified as the optimal predictive model in this study.

**Table 4:** Validation performance comparison between the four models.

**Model/evaluation metrics**	**RMSE**	**MAE**	**MAPE**
Baseline	204.17	146.75	15.62%
ARIMA	300.0303	187.1108	16.38%
LSTM	408.71	226.03	17.21%
ARIMA-LSTM	0.44	0.44	0.08%

RMSE, Root mean square error; MAE, Mean absolute error; MAPE, Mean absolute percentage error.

### 3.6 Forecasting with the optimal model

The ARIMA-LSTM model was employed to forecast the monthly number of foodborne disease cases in Liaoning Province for the year 2025 (Table 5). And compared to the case numbers in 2024, a slight decline is observed.

**Table 5:** Predicted monthly incidence of foodborne diseases in 2025 (case).

**Time**	**Predicted values**
Jan	214.62
Feb	260.84
Mar	462.92
Apr	590.92
May	800.88
Jun	965.11
Jul	2,410.36
Aug	2,651.36
Sept	1,711.15
Oct	941.22
Nov	628.21
Dec	465.05

## 4 Discussion

As one of the most critical global public health challenges, foodborne diseases present extensive coverage, multifactorial influences, and significant management difficulties (Deng et al., 2021). To mitigate their occurrence, comprehensive control measures targeting various influencing factors are essential, along with timely prediction of disease trends to provide evidence-based support for prevention strategies.

The ARIMA model, a classical approach in time series analysis, has been widely applied to short-term forecasting of infectious diseases. In recent years, its application has extended to predicting foodborne diseases (Shao et al., 2021; Liu et al., 2023; Shao and Xia, 2021). For instance, Miao et al. (2021) developed an ARIMA product model to forecast monthly incidence trends of foodborne illnesses. Similarly, Xian et al. (2023) employed SARIMA, Holt-Winters, and exponential models to predict the incidence of foodborne diseases in Nan'an District, Chongqing.

With the continuous advancement of technologies such as computer science and big data, LSTM models have been increasingly applied in the medical field. For example, Fan et al. (2023) developed LSTM neural network, SARIMA, and Holt-Winters models to forecast the incidence trend of hepatitis B (Fan et al., 2023). Khan and Jie (2025) employed a two-stage TSA-LATM model to predict the incidence and mortality rates of cancer. Wan et al. (2023) proposed a combined ARIMA-EEMD-LSTM approach for forecasting the incidence of hand, foot, and mouth disease.

The ARIMA-LSTM hybrid model integrates the strengths of both ARIMA and LSTM approaches, making it particularly suitable for capturing complex temporal dynamics. Owing to its enhanced predictive capability, this model has been increasingly applied in the medical and epidemiological domains. For instance, Jain et al. (2024) utilized the ARIMA-LSTM framework to analyze and forecast the trajectory of COVID-19, while Liu et al. applied it to predict the hand, foot, and mouth disease incidence in Taiwan (Putzu et al., 2024).

In this study, ARIMA, LSTM, and ARIMA-LSTM hybrid models were developed using data from 2015 to 2023, with 2024 data serving as the validation set. While all three models captured the overall trends in 2024, the ARIMA-LSTM model achieved the closest agreement with the observed values, confirming its superiority in predictive performance. However, this model is not without limitations. Although the hybrid structure allows ARIMA and LSTM to complement each other, it does not account for external determinants of foodborne diseases, such as meteorological conditions, geographic distribution, and lifestyle factors, which may contribute to residual prediction errors (Zhang et al., 2024). Future research could therefore enhance the ARIMA-LSTM framework by integrating relevant external variables and fine-tuning the LSTM component to dynamically adapt to variations in the data, thereby improving both accuracy and robustness of predictions.

By examining the incidence patterns from 2015 to 2024 and the predicted cases for 2025, it was observed that foodborne diseases usually peak in summer (July–September). Therefore, during this period, stricter food management and safety inspections in the catering sector are essential. For example, leftovers should be refrigerated promptly, reheated thoroughly before consumption, raw and cooked foods should be stored separately, and untreated water should be avoided. Public health authorities could also issue early warnings and strengthen education campaigns before the summer season. Since older adults and children have weaker immune systems and higher susceptibility, it would be beneficial to enhance food safety training in schools and nursing institutions and to develop dietary guidance tailored for these vulnerable groups. Although external variables were not included in this study, future work could incorporate meteorological, population mobility, and food monitoring data through collaboration with relevant agencies to enhance the model's interpretability and generalizability.

In conclusion, the ARIMA-LSTM model outperformed the ARIMA model and LSTM model in forecasting the incidence of foodborne diseases in Liaoning Province, effectively capturing the trend and providing a valuable reference for developing prevention and control strategies.

## Data Availability

The original contributions presented in the study are included in the article/supplementary material, further inquiries can be directed to the corresponding authors.
